# Drug Repurposing for the Management of Depression: Where Do We Stand Currently?

**DOI:** 10.3390/life11080774

**Published:** 2021-07-30

**Authors:** Hosna Mohammad Sadeghi, Ida Adeli, Taraneh Mousavi, Marzieh Daniali, Shekoufeh Nikfar, Mohammad Abdollahi

**Affiliations:** 1 Toxicology and Diseases Group (TDG), Pharmaceutical Sciences Research Center (PSRC), The Institute of Pharmaceutical Sciences (TIPS), Tehran University of Medical Sciences, Tehran 1417614411, Iran; hosnams1379@gmail.com (H.M.S.); adeli_ayda@yahoo.com (I.A.); st-Mousavi@student.tums.ac.ir (T.M.); M-daniali@student.tums.ac.ir (M.D.); 2 Department of Toxicology and Pharmacology, School of Pharmacy, Tehran University of Medical Sciences, Tehran 1417614411, Iran; 3 Personalized Medicine Research Center, Endocrinology and Metabolism Research Institute, Tehran University of Medical Sciences, Tehran 1417614411, Iran; nikfar_sh@tums.ac.ir; 4 Pharmaceutical Sciences Research Center (PSRC) and the Pharmaceutical Management and Economics Research Center (PMERC), Evidence-Based Evaluation of Cost-Effectiveness and Clinical Outcomes Group, The Institute of Pharmaceutical Sciences (TIPS), Tehran University of Medical Sciences, Tehran 1417614411, Iran; 5 Department of Pharmacoeconomics and Pharmaceutical Administration, School of Pharmacy, Tehran University of Medical Sciences, Tehran 1417614411, Iran

**Keywords:** clinical trials, depression, major depressive disorder, new drugs, repurposing, repositioning, strategies

## Abstract

A slow rate of new drug discovery and higher costs of new drug development attracted the attention of scientists and physicians for the repurposing and repositioning of old medications. Experimental studies and off-label use of drugs have helped drive data for further studies of approving these medications. A deeper understanding of the pathogenesis of depression encourages novel discoveries through drug repurposing and drug repositioning to treat depression. In addition to reducing neurotransmitters like epinephrine and serotonin, other mechanisms such as inflammation, insufficient blood supply, and neurotoxicants are now considered as the possible involved mechanisms. Considering the mentioned mechanisms has resulted in repurposed medications to treat treatment-resistant depression (TRD) as alternative approaches. This review aims to discuss the available treatments and their progress way during repositioning. Neurotransmitters’ antagonists, atypical antipsychotics, and CNS stimulants have been studied for the repurposing aims. However, they need proper studies in terms of formulation, matching with regulatory standards, and efficacy.

## 1. Introduction

Despite the high rate of technological progress and improvements in knowledge of different diseases, the discovery of new medications demonstrated a lower speed [1].

As the regulatory requirements for bringing a new medication to the market is becoming more challenging to meet, medication cost increases globally [2].

Drug repositioning, also called drug repurposing or re-tasking, is a promising strategy to introduce new indications for other therapeutic goals for an available drug in the market [3]. Since the safety profile of these medications was studied thoroughly before, the development of their formulation has been analyzed, and the medicines successfully passed the preclinical and clinical steps, the risk of failure decreases significantly [4]. To apply a drug repositioning strategy, three main steps are needed to be concluded; first, a molecule/substance should be suggested for the mentioned indication; second, preclinical models, including animal and computational models, should be assessed and last the efficacy of medication should be analyzed [5].

As a significant mental disorder, major depressive disorder (MDD) affects approximately 264 million globally [6]. Due to the insufficient therapeutic response of patients to the available medications, the need for new medicines has attracted scientists worldwide [7]. Various mechanisms have been associated with the prevalence of MDD, and these mechanisms directly influence medication selection. Changes in inflammatory biomarkers, neurotransmitters, age-related, and genetic factors are among the mechanisms constituting the indications of medications [8]. Moreover, new antidepressant medications usually act on multiple intra- and extra-cellular markers, indicating their poly-pharmacology indications [9]. This review aims to provide a complete insight into the drug repositioning strategy, especially the candidates that could be beneficial in managing MDD.

## 2. Drug Repurposing

### 2.1. History

Drug repurposing is the procedure of finding new indications for approved or investigational medications [10,11]. Investigational medicines may have had a desirable safety profile in phase I/II clinical trials [12] but never reached the market [11,12] due to reasons unrelated to safety [12], such as lack of efficacy [11]. Drug repurposing can emerge in different forms like repositioning, reformulation, and combination [13,14]. Drug repurposing was serendipitous and accidental in the past; whenever a medication had shown an off-target or a new on-target effect, it was investigated for commercial exploitation. [10]. So far, the most successful repurposed drugs have been found accidentally, and no systematic approach has been involved in the process [10,15]. Sildenafil citrate was an antihypertensive medication that got repurposed as retrospective clinical data analysis showed its positive effect on erectile dysfunction [10,16]. The year sildenafil got marketed by Pfizer for erectile dysfunction under the name Viagra^®^ [17], it held a 47% share of this problem’s market, and the total sales were USD 2.05 billion worldwide [10]. Thalidomide is another well-known instance causing many severe skeletal birth defections in children whose mothers had taken this medication during the first trimester of pregnancy [1,10]. In consequence, thalidomide was withdrawn for four years [10]. In 1964, thalidomide was fortuitously recognized to be effective in erythema nodosum leprosum (ENL) [18]. Decades later, in 1999, thalidomide was discovered to be effective in multiple myeloma [19]. The positive outcome in multiple myeloma led to further derivative developments like lenalidomide [10]. Bupropion, an antidepressant medication got approved by the United States Food and Drug Administration (US-FDA) for smoking cessation [13], botulinum toxin A, the compound used for eye muscle disorders with cosmetic impacts [18] and minoxidil, the antihypertensive medication, became established for pattern hair loss in male and female [17,20] are some instances of well-known repurposed drugs [12]. Iproniazid, an antitubercular compound, was the first medication got reported for its antidepressant effect. This compound showed euphoria, psychostimulation, increased appetite, and improved sleep as the side effects [21]. The story of finding D-lysergic acid diethylamide (LSD) psychedelic effects is another exciting example of serendipitous discovery in the field of psychiatry. LSD was first synthesized in 1938, but it did not show considerable physiological effects in animal testing. LSD’s strong and extraordinary influences on the mind were accidentally discovered for the first time in 1943 [22]. Thirteen medications have been repurposed for depression or bipolar depression treatment by 2017 [13].

### 2.2. Different Types of Drug Repurposing

Drug reformulation, repositioning, and combination are counted as different drug repurposing/repositioning [13,14].

#### 2.2.1. Drug Repositioning

It finds new indications for a medication that already has other therapeutic indications [13,14]. For instance, mifepristone, an anti-progesterone drug with an initial indication for abortion, was experimentally effective in psychotic depression [3,23].

#### 2.2.2. Drug Reformulation

It is about using a medication in a new dosage form [13,14]. The new formulation can both be taken via the same old route or a different route of administration. An example of drug reformulation is formulating ketamine for intranasal and sublingual routes to treat MDD [13].

#### 2.2.3. Drug Combination

It refers to using two or more medications together [13,14] to improve efficacy and safety [13]. For example, using quetiapine and antidepressants in combination leads to the increased effect of antidepressant medications in the elderly suffering from MDD and cerebrovascular deterioration. In addition, taking anti-inflammatory medicines with antidepressants enhances responses to first-line antidepressants [13].

### 2.3. Common Approaches

Before the development stage in drug repositioning, three levels should be considered. The first level is discovering appropriate molecules for the desired indication, wherein most drug repurposing approaches are related to it. The second one is evaluating effects in preclinical experiments. The last is the appraisal of efficacy in phase II clinical trials, given that safety has been approved in phase I clinical trials for the original use [10].

Repositioning approaches are divided into two major groups: computational and experimental strategies [10,12,16]. These are growingly being used together [10,16] and will be separately discussed as follows. Explanations, pros, and cons of the drug repurposing approaches are summarized in Table 1. In addition, examples of each separate approach are indicated in Table 2.

#### 2.3.1. Computational Approaches

Computational approaches consist of data analysis. These data can be obtained from different resources. For example, gene expression, chemical structure [10,12], or electronic health records (EHRs) can all be kinds of data resources [10]. In comparison with experimental approaches, computational approaches have lower expenses and fewer barriers [3].

##### Signature Matching

This method compares exclusive features (signature) of medication with another medication or disease. Three different types of data could be used as resources for extracting medication characteristics: chemical structure, adverse event profiles, proteomics, transcriptomic, and metabolomics, which are explored aptly in the following [10]:

**Transcriptomic**: This technique compares gene expression in a healthy state, disease-associated state, and medication-using state. If a medication can reverse the expression pattern of the genes related to disease phenotype, it will probably also revert the disease phenotype itself [10,12,16,23,24,25].
An example of this approach is that ketamine improves mood by modulating miRNAs like miR-598-5p and miR-451 [13]. Histone deacetylase (HDAC) inhibitors like vorinostat are promising drug repositioning targets for depression, anxiety and schizophrenia treatment due to their role in affecting gene expression [26]. Peroxisome proliferator-activator receptor (PPAR-γ) agonists, especially pioglitazone, have significant antidepressant outcomes in MDD and major depressive episodes of bipolar disorder due to their role in adjusting responsible gene expressions [13]. HMG-CoA reductase inhibitors (statins) are PPAR-α ligands that increase the expression of some neuronal growth factors. Randomized controlled trials have suggested that they possess beneficial effects in combination with selective serotonin reuptake inhibitors (SSRIs) [21].
**Metabolomics:** Metabolomics is the study of all chemical procedures in the body [27]. A drug can be shared between two different disease treatments with similar pathophysiology [5]. This approach helps us to gain a comprehensive idea about the molecular processes involved in disease pathophysiology and finding how close our preclinical models to reality are [28]. Nuclear magnetic resonance and mass spectroscopy are two methods for analyzing the metabolome [29].
**Proteomics:** Most medications apply their therapeutic effects by interacting with protein targets, and it is crucial to understand these interactions for drug development [12].
**Chemical Structure**: In this method, networks are made based on the shared chemical features [10] as similarity in chemical structure may lead to the same biological activity [10,16]. As an example, chlorcyclizine belongs to the phenylpiperazine class. This class includes many antipsychotic and antidepressant medications and chemical structure similarities between these medications and chlorcyclizine make it likely to possess the same effects [26].
**Adverse Event Profiles:** A hypothesis suggests that two different medications showing the same adverse effects might affect a shared target, protein, or pathway [10,16]. As well, a medication’s adverse effect resembling a disease phenotype can imply a shared pathway or physiology between the drug and the illness [10,15,16]. In addition, if two treatments for one disease with different mechanisms demonstrate the same uncommon adverse effect, there may be a shared underlying mechanism that links adverse events and therapeutic effects [30]. Side effects are more helpful in predicting drug indications than chemical structure or protein targets. It is possible to extract adverse events data from chemical structures if a drug has not reached the clinical trial level [31].

Cabergoline, an ergot derivative dopamine agonist, showed delusion adverse events and got recognized to have antidepressant-like effects. Pergolide, another dopamine agonist, demonstrated antidepressant effects in Parkinson’s disease. Modafinil is a narcolepsy medication that may be effective in depression treatment in combination with fluoxetine. Phenytoin, the famous anticonvulsant medicine, can be efficient in depression. This effectiveness is a result of hyperacusis, the phenytoin adverse effect [15].

##### Computational Molecular Docking

The basis of this approach is complementarity between ligand and target [10,23]. In conventional docking, the target involved in the disease is already known, and different medications get tested. In inverse docking, a set of targets are studied to check if they match particular medicines [10]. Computational molecular docking indicates that dextromethorphan shows an antidepressant effect with rapid onset of action during the first administration days due to involving glutamatergic receptors [13]. Cyproheptadine was hypothesized to improve depression based on its potential ability to be a serotonin receptor (5-HT2) antagonist [26]. Computational molecular docking suggests that mecamylamine, a nicotinic receptor antagonist, might be effective in depression treatment [23].

##### Genome-Wide Associated Studies (GWAS)

This method is proceeded on finding genetic variants associated with common diseases and understanding the biology of disease. In addition, these data can result in recognition of shared targets between conditions [10].

GWAS suggest pregabalin, gabapentin, nitrendipine, alizapride, mesoridazine, levonorgestrel, diethylstilbestrol, papaverine, scopolamine, ketoconazole, arcaine sulfate, ifenprodil, cycloserine, risperidone, and sulpiride all can be repurposed for MDD [23].

##### Pathway or Network Mapping

This approach is building networks based on signature matching data, protein interactions [10,25,32], gene expression pattern [10,25], disease pathology, or GWAS data [10] to find similarity or relation between medication and disease [25]. Some disease-associated genes are not appropriate druggable targets. Therefore, constructing and analyzing such networks could be a way to find upstream or downstream genes which can be used for drug repurposing [10]. Pathway mapping based on disease pathogenesis showed that nimodipine, a calcium channel blocker, makes antidepressants effective in old patients suffering from vascular depression. Scopolamine, the muscarinic antagonist, possesses rapid antidepressant effects since the cholinergic system is responsible for the pathogenesis of mood disorders [13]. Cannabidiol is a propitious agent for MDD due to its effects on involved pathways in this disorder. Sho-saiko-to, a traditional Chinese medicine, showed antidepressant effects in mice upon its influence on the serotonergic system in the central nervous system. Medications that inhibit p38 mitogen-activated protein (p38-MAPK) signaling pathways such as neflamapimod may have desirable effects on depression as the p38-MAPK pathway is engaged in many cellular processes, especially neuro-inflammation. Spermine is useful in treatment-resistant depression (TRD), as it is a glutamatergic receptor modulator. N-acetyl-l-cysteine (NAC), the glutathione precursor, positively affects the different mechanisms involved in depression and is a beneficial nutraceutical for adding to antidepressant medications in MDD treatment [21].

#### 2.3.2. Experimental Approaches

##### Retrospective Clinical Analysis

The main idea of this approach is reviewing and extracting valuable data from different resources such as EHRs, post-marketing surveillance, and clinical trials. This process would result in repositioning and using a medication for a dissimilar indication or finding an indication for a drug that had failed for its initial purpose [10].

**EHRs**: EHRs data are subdivided into structured (diagnosis and pathophysiology data, laboratory test results, and medication prescriptions) and unstructured (patients’ symptoms reports and imaging data) groups [10].
Analyzing data gained from the national health insurance of Taiwan research database showed metformin is a promising target for drug repositioning for depression and anxiety [26]. The results of observational or case-control studies indicated a lower risk of MDD in patients using angiotensin-converting enzyme inhibitors (ACEIs) like telmisartan in comparison with other antihypertensive medications. Case reports also demonstrated that pramipexole, a relatively new dopamine receptor agonist, has potential effects in treating MDD. An observational study on 82,643 women revealed the relation between higher flavonoid intake and lower risk of MDD. Last but not least, a meta-analysis of 17 observational studies suggested the association between depression and zinc deficiency [21].
**Post-Marketing Surveillance and Clinical Data**: Retrospective clinical data analysis showed that using anti-inflammatory medications, especially celecoxib, with antidepressants improves the responses to first-line antidepressants. Clinical data claims that valproic acid enhances the effects of antidepressants in resistant depression patients. Based on clinical data, quetiapine co-administered with antidepressants improves the outcomes in patients with MDD and cerebrovascular deterioration [13]. Reviewing prior literature has shown that phenothiazines have anti-depressive effects resembling tricyclic antidepressants. Atypical antipsychotics can also help treat depression as adjunctive or primary therapy based on a meta-analysis [28]. Taking antidepressants combined with zinc caused decreased depressive symptoms than antidepressants alone in randomized control trials [21].

##### Novel Sources

This approach consists of three methods. The first one is using immortalized human cancer cell lines (CCLs) for screening different compounds to examine if pharmacological and genetic data match. The second method links EHR data to DNA biobanks; thereby, identifying the association between the patient’s genome and the patient’s illness. The third novel resource is patients’ online self-reported data about their condition while taking medicine [10].

##### Binding Assays

Binding assessments help us realize target and ligand interactions. For example, affinity chromatography, mass spectroscopy, and cellular thermos ability assay (CETSA) techniques are three methods used in this approach [10].

##### Phenotypic Screening

This approach attempted to identify compounds showing effects of disease consequences in model systems without any earlier information about targets they affect [10].

**Table 1:** Explanations, advantages and disadvantages of approaches commonly used for drug repurposing.

				Explanation		Pros and/or Cons/Ref
Common Approaches	Computational Approaches	Signature Matching	Transcriptomic, Metabolomics, and Proteomics	Transcriptomic	Comparison of Gene Expression in Healthy, Disease-Associated and Medication-Using State	Pros: finding new targets or off-target effects for existing medications [10], finding the new mechanism of action for drugs, involving more genetic level mechanisms in comparison to knowledge-based methods [25,32], low costs, public access to databases [23] Cons: medication-target genes not getting expressed in altered patterns and not being detectable [33]
				**Metabolomics**	Recognizing potentially druggable targets in different diseases	Pros: involving more molecular level mechanisms in comparison to knowledge-based methods [25]
				**Proteomics**	Applying interaction between medication and proteome	Pros: gaining information about safety, probable medication toxicity, mode of action of small molecule medications [12], involving more genetic and/or molecular level mechanisms in comparison to knowledge-based methods, finding new mechanisms of action [25,32]
			**Chemical structure**	Developing networks based on shared chemical features		Cons: the difference between actual results and expectations, variety of physiological effects despite structure resemblance [10], possibility of happening alterations in structure due to biological activity [16]
			**Adverse event profile**	Finding shared targets, proteins, or pathways affected by different medications showing the same adverse effects		Pros: unlike animal models, both therapeutic and adverse effects are observable in humans [15] Cons: problems in extracting information on medication package inserts, lack of proper adverse event profile and causality assessment [10]
		**Computational molecular**	Testing different medications on a known target (conventional docking) or checking different targets			Pros: the ability to test all compounds with recognized structure [25]
		**Docking**	Match a particular medication (inverse docking) based on complementarity between ligand on target			Cons: difficulty in providing a 3D image of G-protein receptors, lack of eligible database providing appropriate information about targets’ structures, the difference between actual affinity between ligand and target and virtual results, different outcomes of different software packages [10], impossibility of identifying unknown mechanism beyond the known target in conventional docking [25]
		**Genome-wide associated data (GWAS)**	Finding genetic variants associated with common diseases and understanding the biology of disease			Pros: advances in technology, the accomplishment of the HGP, reduction in genotyping costs Cons: need for studies to assure in which direction gene variants affect disease, difficulty in identification responsible gene or gene variants due to linkage disequilibrium in gene-rich loci, not providing a comprehensive insight into disease pathophysiology, lack of definite knowledge of the human genome and the possibility of new human genes discovery [10], disease’s complicated genetic basis due to polygenic nature of illness [25]
		**Pathway or network mapping**	Building networks for disease or medication based on signature matching data, gene expression pattern, disease pathology, GWAS data, or protein interactions			Pros: narrowing the range of molecules from a large number to few targets [12]
	**Experimental approaches**	**Retrospective clinical analysis**	Electronic health records (EHRs)	Reviewing and extracting data from structured (diagnosis and pathophysiology data, laboratory test results, and medication prescriptions) and unstructured (of patients’ symptoms reports and imaging data)		Pros: easy access to different resources such as WHO or FAERS database and ability to analyze these data [10], discovering adverse events that were not noticed during drug development by applying Natural Language to EHRs [2] Cons: ethical and legal obstacles, challenges in extracting unstructured data [1]
			Post-marketing surveillance and clinical data	Analyzing post-marketing surveillance and clinical data to extract information		Cons: restriction in accessing to these data due to commercial and ethical reasons [1]
		**Novel sources**	Using immortalized human CCLs for screening different compounds, linking EHR data to DNA bank to identify an association between patients’ genome and patients’ illness, using patients’ online self-reported data while taking medicine			Pros: development of sequencing technologies, which helps ones collect more thorough information on each patient’s genetics (2), acceleration in the drug discovery process, reduction in research costs, increase in patient involvement, ability to assess the effectiveness of the in-use medication (3) Cons: Happening alterations that make in vitro results better (1), challenges in using big data and technology for analysis (2), bias in collecting data, threat in patients’ safety in case of self-prescription (3) [10]
		**Binding assays**	Realizing interactions between target and ligand by using different methods as chromatography and mass spectroscopy			-
		**Phenotypic screening**	Identifying compounds showing disease consequences related effects in model systems, without any prior information about targets			Pros: testing many medications for a therapeutic effect over a complete range of concentration [34], high flexibility for administration to numerous drugs or diseases [32] Cons: not reaching a complete picture by in vitro assays [33]

CCLs: Cancer cell lines: FAERS: Food and Drug Administration adverse event reporting system; HGP: Human genome project; Ref: Reference; WHO: World Health Organization.

### 2.4. Advantages

Drug discovery is a high-cost and lengthy process. Bringing a new medication to market takes 13–15 years and costs USD 2–3 billion [10,21,25]. Although the expenses are increasing, the number of approved medications has remained constant or even decreased through the past years [22,25]. Moreover, demands in therapeutic fields are growing, and traditional drug discovery cannot answer these needs [13]. In the case of psychiatric medications, it should be noted that this field has not been developed enough over time [26]. Drug repurposing is cost-effective and reduces the time taken to get a new medication to market [10,11,21] as it costs on average USD 300 million and takes about 6.5 years [10,21]. The preclinical tests [10,16] and phase I and II clinical trials can be skipped in drug repurposing if these steps have already passed for other indications and safety has been approved [10]. This is why drug repurposing may shorten the time needed and expenses as mentioned above [1,10].

Furthermore, if the formulation is appropriate for the new indication, there is no need for formulation development. This can also be another helping hand for reaching the stated aims. Another great pro of drug repurposing is the lowered risk of failure due to approved sufficient safety [10,16]. At last, drug repurposing may uncover novel targets or pathways in treating a disease, which can be exploited further [10].

### 2.5. Barriers

**Figure 1:**
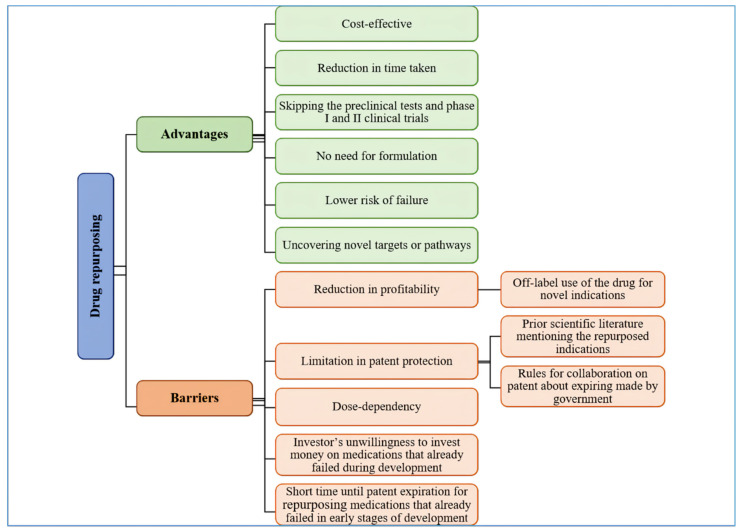
Advantages and barriers of drug repurposing.

Although toxicity and safety are not obstacles in drug repurposing, some barriers lead to failures, such as patent consideration, regulatory issues, and organization hurdles. In brief, many of the repurposed uses are already mentioned in the prior scientific literature or clinical data leading to limitations in patent protection. In addition, when an available generic formulation gets repurposed for a new indication, profitability reduces. This reduction happens due to off-label using the medication for novel indications. Governments make some rules for collaboration on patents that are near expiring to save public benefit. Creating a new formulation or dosage forms, developing new derivatives with the same therapeutic effects, or presenting medication in a new geographic region market are strategies for making a profit from the repurposed drugs [10]. Another trouble is that the effect of the medication is dependent on its dose. Therefore, it is necessary to identify the appropriate dose for novel indications during clinical trials [16]. Investments might be another obstacle in repurposing medications that have already failed during the drug development process. This trouble happens due to investors’ unwillingness as they see medication’s failure. In addition, medicines that failed in later stages of drug development have less time until patent expiration for repurposed try. Designing parallel development processes for different indications can lower the risk of failure [1] (Figure 1).

**Table 2:** Examples of repurposed/suggested repurposing medications for different types of depression with their repurposing approach.

Medication Name	Repurposing Approach/Ref	New Indication Suggested by Article, or Investigational/FDA Approved/Ref
Atypical antipsychotics Quetiapine Aripiprazole Brexpiprazole	Retrospective clinical analysis (PM surveillances and CD) [13]	MDD, BP1 depressive episodes (FA) [8]
	NA	MDD (FA) [9]
	NA	MDD (FA) [35]
Mecamylamine	Computational molecular docking [23]	Depression (SA) [23], MDD (INV) [17,19,20,36,37]
Cyproheptadine	Computational molecular docking [26]	NA
Dextromethorphan	Computational molecular docking [13]	MDD (INV) [38,39,40,41]
Pregabalin	GWAS [23]	MDD (SA) [23]
Gabapentin	GWAS [23]	MDD (SA) [23]
Cycloserine	GWAS [23]	BP depression (INV) [42], MDD (SA, INV) [23,36]
Risperidone	GWAS [23]	Extrapyramidal symptoms, suicidal ideation (INV) [43]
Cannabidiol	Pathway or network mapping [21]	MDD (SA) [21]
N-acetyl-l-cysteine	Pathway or network mapping [21]	MDD (SA) [21]
Sho-saiko-to	Pathway or network mapping [21]	NA
Spermine	Pathway or network mapping [21]	TRD (SA) [21]
Nimodipine	Pathway or network mapping [21]	Vascular depression in old patients (SA) [13]
Scopolamine	GWAS, Pathway or network mapping [13]	MDD (INV) [44,45]
Anti-inflammatory medications	Retrospective clinical analysis (PM surveillances and CD) [13]	NA
Valproic acid	Retrospective clinical analysis (PM surveillances and CD) [13]	Resistant depression (SA) [13]
Zinc	Retrospective clinical analysis (PM surveillances and CD) [21]	MDD (INV) [46]
Pramipexole	Retrospective clinical analysis (EHRs) [21]	MDD (SA, INV) [21,47]
Telmisartan	Retrospective clinical analysis (EHRs) [21]	MDD (SA) [21]
Metformin	Retrospective clinical analysis (EHRs) [26]	MDD (INV) [48]
Phenothiazines	Retrospective clinical analysis (PM surveillances and CD) [26]	NA
Cabergoline	Signature matching (adverse event profile) [15]	NA
Modafinil	Signature matching (adverse event profile) [15]	MDD (INV) [49], depressive episode in BP1 disorder (INV) [50], major depressive episode in BP1 disorder (INV) [51]
Pergolide	Signature matching (adverse event profile) [15]	NA
Phenytoin	Signature matching (adverse event profile) [15]	NA
Chlorcyclizine	Signature matching (chemical structure) [26]	NA
Vorinostat	Signature matching (transcriptomic) [26]	Depression, anxiety, schizophrenia (SA) [26]
Statins	Signature matching (transcriptomic) [21]	NA
Ketamine (esketamine)	Signature matching (transcriptomic) [13]	MDD and bipolar depression, Depressive symptoms in adults with MDD with acute suicidal ideation or behavior (FA) [52]
Pioglitazone	Signature matching (transcriptomic) [13]	MDD, a major depressive episode in bipolar disorder (SA) [13]

BP: Bipolar; CD: Clinical data; FA: FDA approved; GWAS: Genome-wide associated studies; INV: Investigational; MDD: Major depressive disorder; NA: Not available; PM: Post-marketing; Ref: Reference; SA: Suggested by articles; TRD: Treatment-resistant depression.

## 3. Management of Depression

**Figure 2:**
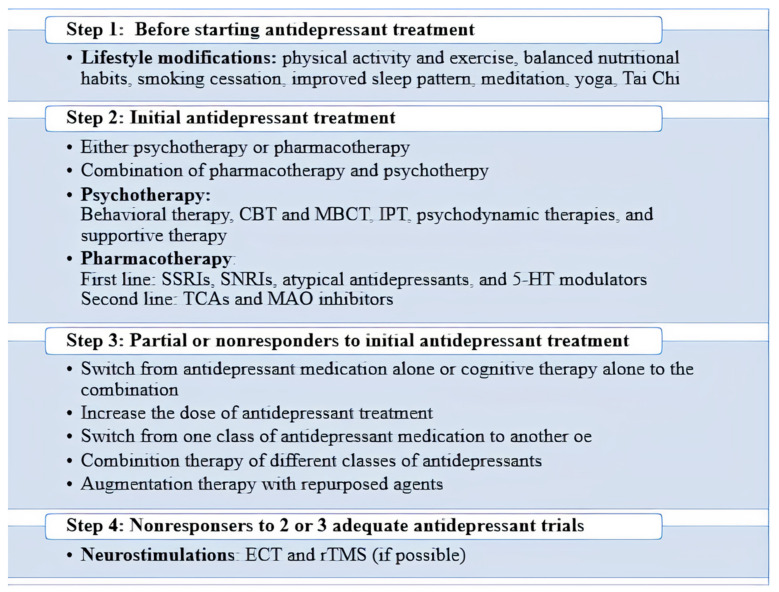
MDD management steps. Abbreviations: CBT: Cognitive behavioral therapy; IPT: Interpersonal psychotherapy; MAO: Monoamine oxidase; MBCT: Mindfulness-based cognitive therapy; MDD: Major depressive disorder; SSRI: Selective serotonin reuptake inhibitors.

In order to manage a patient diagnosed with MDD, two or three main options, including psychotherapy, pharmacotherapy, and somatic interventions, exist. Some guidelines suggest that those with moderate to severe depression would benefit from both psychotherapy and pharmacotherapy. In a mildly depressed person, treatment could be initially based on psychotherapy, and if needed, switching to medication could be applied after weeks [17]. Physical activity and exercise, balanced nutritional habits, improved sleep patterns, etc., can impact mental health and might be beneficial towards depressive disorders. Training like meditation, yoga, Tai chi, or daily journaling events is another helpful way to reduce stress, leading to the improved mental condition [53] (Figure 2).

In mild-to-moderate depression, psychotherapy has proved to be adequate and comparable to pharmacological therapies. Many experts suggest different types of psychotherapy like cognitive-behavioral therapy, behavioral activation therapy, and interpersonal psychotherapy. However, for severe forms of depressive disorder, antidepressant drugs have appeared to be much more effective by possessing a more rapid onset of action. Furthermore, psychotherapy is often used in those who have shown a response to antidepressants to prevent its relapse. Overall, the combination of both therapies is suggested and has been demonstrated to be more effective to either alone [48]. It is shown that the beneficial effects of different psychotherapy methods can last for at least one year after the treatment process. However, many people refuse this choice due to its high costs, lack of time, and the recent issue of Coronavirus disease-2019 (COVID-19). As solutions, attending some group psychotherapies to reduce the costs, setting online or over the phone sessions are valued, although all patients still do not believe in such ways [17]. Somatic intervention is another non-pharmacological option for the treatment of MDD. Electroconvulsive therapy (ECT) and repetitive transcranial magnetic stimulus (rTMS) are some noninvasive examples suggested to be beneficial for patients who have already failed at least one antidepressant trial. Vagus nerve stimulation is a US-FDA-approved surgical and invasive procedure for the management of TRD, which of course, carries its risks [53].

### 3.1. Pharmacotherapy

After a patient is diagnosed with MDD, a psychiatrist can start pharmacologic treatment to symptom remission. Antidepressants take approximately 3–4 weeks to exert their effects, although there is always the risk of relapse or recurrence of mood episodes even after the therapy. Choosing the first-line treatment for a patient depends on multiple factors, including age, concurrent medical conditions or psychiatric state, adverse effect profiles of the drug and its interactions, ease of access, cost, convenience and patient’s preference, safety in overdose, etc. Another essential issue is the patient’s initial responses to antidepressants (if taken) and the family history [53].

#### 3.1.1. Current Antidepressant Medications

Today, according to Katzung Basic and Clinical Pharmacology’s last edition, five (or six) main antidepressant categories exist and exert their effects through various molecular targets and mechanisms of action. These categories include selective serotonin reuptake inhibitors (SSRIs), serotonin-norepinephrine reuptake inhibitors (SNRIs) with two subgroups of selective serotonin-norepinephrine reuptake inhibitors, and tricyclic antidepressants (TCAs), 5-HT2 receptor modulators, tetracyclic and unicyclic antidepressants, monoamine oxidase inhibitors (MAOIs) [54]. The sixth category consists of some various antidepressant medications such as tianeptine (alpha-amino-3-hydroxy-5-methyl-4-isoxazole propionic acid (AMPA) and N-methyl-D-aspartate (NMDA) receptors modulator) [55], reboxetine (a selective inhibitor of norepinephrine reuptake), agomelatine (an agonist for melatonin (MT1 and MT2) and serotonin (5-HT2C) receptors) [19], ademetionine (a major methyl donor, required for the synthesis of several neurotransmitters) [56] and agmatine (an NMDA receptor inhibitor) [57], etc.

#### 3.1.2. Repurposed Drugs for MDD

Plenty of repurposed agents for depression are studied or have gotten US-FDA approval for use in the clinic from different pharmacological categories. These categories vary from some central nervous system (CNS)-related medications like the second generation (atypical) antipsychotics, NMDA receptor antagonists and anesthetics, GABA receptor modulators, dopamine agonists, anticholinergic agents, CNS stimulants, anticonvulsant agents, histamine antagonists and ergot derivatives to even some unrelated ones such as thyroid products, antidiabetic agents, anti-inflammatory agents, antibiotics, HMG-CoA reductase inhibitors, calcium channel blockers, angiotensin-converting enzyme inhibitors, antineoplastic agents and some nutritional supplements. These agents exert their antidepressant effects through various pathways due to their pharmacological category. The complete list of repurposed drugs for MDD with a particular focus on their mechanism of action, significant adverse effects, contraindications, and dosages (if available) are provided in Table 3. Moreover, related clinical trials studying their effects on MDD in real-world settings are summarized in Table 4.

## 4. Conclusions

MDD, as a severe mental disorder declining the quality of life of the patient, requires on-time screening and management. Psychotherapy, pharmacotherapy, and somatic interventions are among the suggested managements. However, due to the incomplete response of the patients to the approved medications, physicians tend to prescribe medications on their off-label use. Hence, a great need for a drug repositioning method took place for the MDD management medications. Drug repositioning is a cost-effective method that decreases the required time to introduce medicine to the market.

Moreover, as there are available data on the safety profile of the medications, the risk of failure decreases significantly, and this method is capable of uncovering novel targets to treat a disease. Various pharmacological categories, including neurotransmitters’ antagonists, atypical antipsychotics, and CNS stimulants, have been studied for the repurposing aims. However, proper studies on the formulation, regulatory, and efficacy of the medication are required to better this approach.

## Figures and Tables

**Table 3:** Pharmacological and clinical profile of repurposed medications for MDD.

Medication/Bioactive Compound Brand Name/ Ref	US-FDA Approval	Pharmacological Category/Mechanism of Action	Dosage	Significant Adverse Effects	Contraindications
**Aripiprazole** Abilify^®^ [58]	Bipolar disorder, irritability associated with autistic disorder, MDD, TRD, schizophrenia, Tourette disorder	Partial agonist at the D_2_ and 5-HT_1A_ receptors, an antagonist at the 5-HT_2A_/Second-generation (atypical) antipsychotic	MDD and TRD as an adjunctive treatment. Oral: 2 to 5 mg/day	>10%: decreased HDL-C, increased LDL-C, increased serum cholesterol, increased serum TG, weight gain, akathisia, headache, increased serum glucose, constipation, nausea, and vomiting	Hypersensitivity to aripiprazole or any component of the formulation
			Increase dose based on response in 5 mg increments up to a maximum of 15–20 mg/day		
**Brexanolone** Zulresso^®^ [59]	Postpartum depression (PPD) in adults	The mechanism of action is not fully understood, but is thought to be related to its positive allosteric modulation of GABA_A_ receptors/GABA_A_ receptor-positive modulator	Postpartum depression. IV: 0 to 4 h: 30 mcg/kg/h 4 to 24 h: 60 mcg/kg/h 24 to 52 h: 90 (or 60) mcg/kg/h 52 to 56 h: 60 mcg/kg/h 56 to 60 h: 30 mcg/kg/hour	>5%: sedation/somnolence, dry mouth, loss of consciousness, and flushing/hot flush	No contraindications were listed in the US-FDA monograph
**Brexpiprazole** Rexulti^®^ [60]	MDD, schizophrenia	Partial agonist activity for 5-HT_1A_ and D_2_ receptors and antagonist activity for 5-HT_2A_ receptors/Second-generation (atypical) antipsychotic	MDD as an adjunct therapy to antidepressants. Oral: 0.5 mg or 1 mg once daily; titrate to 1 mg once daily, followed by 2 mg once daily; maximum daily dose: 3 mg	>10%: increased serum TG, weight gain, akathisia	Hypersensitivity (e.g., anaphylaxis, facial swelling, rash, urticarial) to brexpiprazole or any component of the formulation
**Cabergoline** Dostinex^®^ [61]	Hyperprolactinemic disorders	Long-acting dopamine receptor agonist with a high affinity for D2 receptors, a potent 5-HT2B-receptor agonist/Ergot derivative	NA	Headache, dizziness, nausea	Known hypersensitivity to cabergoline, ergot derivatives, or any component of the formulation, uncontrolled hypertension; history of cardiac valvular disorders, history of pulmonary, pericardial, or retroperitoneal fibrotic disorders
**Cannabidiol** Epidiolex^®^ [62]	Treatment of seizures associated with Lennox-Gastaut syndrome or Dravet syndrome in patients with 2 years of age and older	The precise mechanisms of its anticonvulsant effect in humans are unknown; however, it does not appear to exert its effects through interaction with cannabinoid receptors/Anticonvulsant, cannabinoid	NA	>10%: somnolence, decreased appetite, diarrhea, transaminase elevations, fatigue, malaise and asthenia, rash, insomnia, sleep disorder and poor quality sleep, infections	Hypersensitivity to cannabidiol or any of the ingredients in Epidiolex^®^
**Celecoxib** CeleBREX^®^ [63]	Acute pain, ankylosing spondylitis, juvenile idiopathic arthritis, osteoarthritis, primary dysmenorrhea, rheumatoid arthritis	Inhibits prostaglandin synthesis by decreasing COX-2 enzyme/Analgesic, nonopioid, NSAID, COX-2 selective	NA	Serious cardiovascular thrombotic events, myocardial infarction, and stroke increased the risk of serious gastrointestinal adverse events, including bleeding, ulceration, and perforation of the stomach or intestines	Hypersensitivity to celecoxib, sulfonamides, aspirin, other NSAIDs, or any component of the formulation
**Cycloserine** Seromycin^®^ [64]	Tuberculosis, UTI	Inhibits bacterial cell wall synthesis by competing with amino acid (D-alanine) for incorporation into the bacterial cell wall/Antibiotic, antitubercular agent	NA	Cardiac arrhythmia, cardiac failure, coma, confusion, dizziness, drowsiness, dysarthria, headache, hyperreflexia, paresis, paresthesia, psychosis, restlessness, seizure, vertigo, skin rash, cyanocobalamin deficiency, folate deficiency, increased liver enzymes, hypersensitivity reaction, tremor	Hypersensitivity to cycloserine or any component of the formulation, epilepsy, depression, severe anxiety, or psychosis, severe renal insufficiency, excessive concurrent use of alcohol
**Cyproheptadine** Euro-Cyproheptadine^®^, PMS-Cyproheptadine^®^ [65]	Allergic conditions	Potent antihistamine and serotonin antagonist with anticholinergic effects competes with histamine H1 receptors/First, generation histamine H1 antagonist, a piperidine derivative	NA	Extrasystole, hypotension, palpitations, tachycardia, ataxia, chills, confusion, dizziness, drowsiness, euphoria, excitement, fatigue, hallucination, headache, hysteria, insomnia, irritability, nervousness, neuritis, paresthesia, restlessness, sedation, seizure, vertigo, etc.	MAO inhibitor therapy, close angle glaucoma, stenosing peptic ulcer, symptomatic prostatic hypertrophy, bladder neck obstruction, pyloroduodenal obstruction, elderly, debilitated patients
**Dextromethorphan** Buckleys cough^®^, Creomulsion adult^®^, Cough DM^®^, Delsym^®^, etc. [66]	Cough suppressant	Decreases the sensitivity of cough receptors and interrupts cough impulse transmission by depressing the medullary cough center through sigma receptor stimulation/Antitussive, NMDA receptor antagonist	NA	Dizziness, drowsiness, nervousness, restlessness, gastrointestinal distress, nausea, stomach pain, vomiting	Concurrent administration with or within two weeks of discontinuing MAO inhibitor
**Esketamine** Spravato^®^ [52]	TRD, MDD with suicidality	Nonselective, noncompetitive NMDA receptor antagonist/NMDA receptor antagonist	TRD. Intranasal: induction: 56 mg (may increase to 84 mg) twice weekly for 4 weeks Maintenance: on week 5, the previously established dose (56 or 84 mg) once weekly—on week 9 and onward continue effective dose (56 to 84 mg) once weekly or every 2 weeks MDD with suicidality. Intranasal: 84 mg twice weekly for 4 weeks; may reduce dosage to 56 mg twice weekly	>5%: dissociation, dizziness, nausea and vomiting, sedation, vertigo, hypoesthesia, anxiety, lethargy, increased blood pressure, feeling drunk	Hypersensitivity to esketamine, ketamine, or any of the excipients, Aneurysmal vascular disease or arteriovenous malformation, intracerebral hemorrhage
**Etanercept** Enbrel^®^, Erelzi^®^, Brenzys^®^ [67]	Ankylosing spondylitis, plaque psoriasis, polyarticular juvenile idiopathic arthritis, psoriatic arthritis, rheumatoid arthritis	Binds TNF-α and blocks its interaction with cell surface receptors/Antirheumatic, disease-modifying, TNF-α blocking agent	NA	>10%: skin rash, diarrhea, positive ANA titer, antibody development, infection, injection site reaction, respiratory tract infections	Sepsis, hypersensitivity to etanercept, or any component of the formulation
**Gabapentin** Fanatrex FusePaq^®^, Gralise^®^, Gralise Starter^®^, Neuraptine^®^, Neurontin^®^ [68]	Postherpetic neuralgia, seizures	Modulates the release of excitatory neurotransmitters which participate in epileptogenesis and nociception/Anticonvulsant, GABA analog	NA	>10%: dizziness, drowsiness, ataxia, fatigue, viral infections	Hypersensitivity to gabapentin or any component of the formulation
**Infliximab** Avsola^®^, Inflectra^®^, Remicade^®^, Renflexis^®^ [69]	Ankylosing spondylitis, Crohn disease, plaque psoriasis, psoriatic arthritis, rheumatoid arthritis, ulcerative colitis	Binds to human TNF-α and interfere with endogenous TNF-α activity/Antirheumatic, disease-modifying, gastrointestinal agent, immunosuppressant agent, monoclonal antibody, TNF-α blocking agent	NA	>10%: abdominal pain, nausea, anemia, increased ALT, antibody development, increased ANA titer, abscess, infection, headache, cough, pharyngitis, sinusitis	Hypersensitivity to infliximab, murine proteins, or any component of the formulation
**Ketamine** Ketalar^®^ [70]	Induction and maintenance of general anesthesia	Noncompetitive NMDA receptor antagonist (blocks glutamate)/General anesthetic	Depressive episodes and MDD. IV: 0.5 mg/kg administered over 40 min; 1 to 3 times weekly; may increase dose to 0.75 to 1 mg/kg. Treatment up to 6 weeks (optimal duration of therapy is unknown)	>10%: prolonged emergence from anesthesia (includes confusion, delirium, dreamlike state, excitement, hallucinations, irrational behavior, vivid imagery)	Hypersensitivity to ketamine or any component of the formulation, conditions in which an increase in blood pressure would be hazardous
**Liothyronine** Cytomel^®^, Triostat^®^ [71]	Thyroid disorders, a myxedema coma	Manufactured form of the thyroid hormone triiodothyronine (T_3_) and exerts its many metabolic effects through control of DNA transcription and protein synthesis/Thyroid product	Antidepressant augmentation. Oral: 25 mcg/day; may be increased to 50 mcg/day after ~1 week. Dose ranges of 20 to 62.5 mcg/day have been studied in clinical trials.	1% to 10%: cardiac arrhythmia, tachycardia, hypotension, myocardial infraction	Hypersensitivity to liothyronine sodium or any component of the formulation, uncorrected adrenal insufficiency, untreated thyrotoxicosis, concurrent use with artificial rewarming of the patient
**Lisdexamfetamine** Vyvanse^®^ [72]	ADHD, binge eating disorder	Converts to the active component dextroamphetamine, a noncatecholamine, sympathomimetic amines that cause a release of dopamine and NE from their storage sites/Central nervous system stimulant	NA	>10%: insomnia, decreased appetite, xerostomia, upper abdominal pain	Hypersensitivity to amphetamine products or any component of the formulation, concurrent use of MAO inhibitor, or within 14 days of the last MAO inhibitor dose
**Lithium** Lithobid^®^ [73]	Bipolar disorder	Influence the reuptake of serotonin and/or NE and inhibit 2nd messenger systems involving phosphatidylinositol cycle, increasing glutamate clearance, enhancing the expression of neurotrophic factors (BDNF)/Antimanic agent	MDD and TRD as an adjunctive treatment. Oral: 300 to 600 mg/day in 1 to 2 divided doses; may increase every 1 to 5 days to a target dose of 600 mg to 1.2 g/day in divided doses. Clinical improvement may take up to 6 weeks.	The most significant adverse reaction is lithium toxicity; signs: diarrhea, vomiting, drowsiness, muscular weakness, and lack of coordination	Hypersensitivity to lithium or any component of the formulation
**Metformin** D-Care DM2^®^, Fortamet^®^, Glucophage^®^, Glumetza^®^, Riomet^®^ [74]	Type 2 diabetes mellitus	Decreases hepatic glucose production, reduces intestinal absorption of glucose and improve insulin sensitivity, increases peripheral glucose uptake and utilization/Antidiabetic agent, biguanide	NA	>10%: diarrhea, flatulence, nausea and vomiting, dyspepsia, abdominal pain, lactic acidosis, vitamin B12 deficiency	Hypersensitivity to metformin or any component of the formulation, severe renal dysfunction, metabolic acidosis
**Mecamylamine** Vecamyl^®^ [75]	Hypertension	Inhibits acetylcholine at the autonomic ganglia/Ganglionic blocking agent	NA	Orthostatic hypotension, syncope altered mental status, convulsions, fatigue, paresthesia, sedation decreased libido, anorexia, constipation, glossitis, intestinal obstruction, nausea, vomiting, xerostomia, urinary retention, tremor, weakness, blurred vision, mydriasis, pulmonary edema, pulmonary fibrosis	Hypersensitivity to mecamylamine or any component of the formulation; mild, moderate, labile hypertension, coronary insufficiency or recent myocardial infarction, uremia, glaucoma, organic pyloric stenosis, coadministration with antibiotics or sulfonamides
**Minocycline** CoreMino^®^, Minocin^®^, Minolira^®^, Solodyn^®^, Ximino^®^ [76]	Different types of infections, acne, etc.	Inhibits bacterial protein synthesis by binding with the 30S and possibly the 50S ribosomal subunit/Antibiotic, tetracycline derivative	NA	1% to 10%: pruritus, urticaria, dizziness, fatigue, malaise, drowsiness, arthralgia, tinnitus	Hypersensitivity to minocycline, other tetracyclines, or any component of the formulation
**Modafinil** Provigil^®^, Alertec^®^ [77,78]	Narcolepsy, obstructive sleep apnea, shift work sleep disorder	Increase dopamine in the brain by blocking dopamine transporters/Central nervous system stimulant	Antidepressant augmentation for MDD. Oral: 100 mg/day for 3 to 7 days, then increase to 200 mg/day; further, adjust dose based on response and tolerability up to 400 mg/day.	Headache, nausea, nervousness, rhinitis, diarrhea, back pain, anxiety, insomnia, dizziness, dyspepsia	Contraindicated in patients with known hypersensitivity to modafinil, armodafinil or its inactive ingredients
**N-acetyl cysteine** Acetadote^®^, Cetylev^®^, Parvolex^®^ [79]	Acetaminophen overdose, mucolytic	Restoring hepatic glutathione, serving as a glutathione substitute, exerts mucolytic effects through its free sulfhydryl group, which opens up the disulfide bonds in the mucoproteins/Antidote, mucolytic agent	NA	>10%: autoimmune disease, anaphylactoid reaction	Hypersensitivity to acetylcysteine or any component of the formulation
**Nimodipine** Nymalize^®^, Nimotop^®^ [80]	Subarachnoid hemorrhage	Inhibits calcium ion from entering the slow channels/Calcium channel blocker, dihydropyridine	NA	1% to 10%: decrease blood pressure, bradycardia, headache, nausea	Concomitant use with potent CYP3A4 inhibitors
**Olanzapine** ZyPREXA^®^ [81]	Acute agitation/aggression associated with psychiatric disorders, bipolar disorder, unipolar MDD, TRD, schizophrenia	Combination of dopamine and serotonin type 2 receptor antagonism/Antimanic agent, second-generation (atypical) antipsychotic	MDD and psychotic depression as an adjunctive therapy. Oral: 5 mg once daily; may increase the dose in increments of 5 mg up to 20 mg/day. TRD as an adjunctive therapy. Oral: 5 mg once daily; may increase dose up to 20 mg/day.	Postural hypotension, constipation, weight gain, dizziness, personality disorder, akathisia, sedation, headache, increased appetite, abdominal pain, pain in extremity, fatigue, dry mouth, asthenia, drowsiness, tremor	No contraindication with ZyPREXA^®^ monotherapy, caution in combination therapy with fluoxetine, lithium or valproate
			A fixed-dose of olanzapine/fluoxetine combination may be used instead of separate components.		
**Pergolide** Permax^®^, Prascend^®^ [82]	Adjunctive treatment to levodopa/carbidopa in the management of the signs and symptoms of Parkinson’s disease	Potent dopamine receptor agonist/Ergot derivative	NA	Dyskinesia, hallucinations, somnolence, insomnia, nausea, constipation, diarrhea, dyspepsia, rhinitis	Hypersensitive to pergolide mesylate or other ergot derivatives
**Pioglitazone** Actos^®^ [83]	Type 2 diabetes mellitus	Improving target cell response to insulin, a potent and selective agonist for PPARγ/Antidiabetic agent, thiazolidinedione	NA	>10%: edema, hypoglycemia, upper respiratory tract infection	Hypersensitivity to pioglitazone or any component of the formulation, NYHA class III/IV heart failure
**Phenytoin** Dilantin^®^, Phenytek^®^ [84]	Seizures (non-emergent use), status epilepticus	Stabilizes neuronal membranes and decreases seizure activity by modulating efflux or influx of sodium, shortens action potential in the heart/Anticonvulsant, hydantoin	NA	Nystagmus, ataxia, slurred speech, decreased coordination, somnolence, mental confusion	Hypersensitivity to phenytoin, other hydantoins, or any component of the formulation, concurrent use of delavirdine, history of prior acute hepatotoxicity attributable to phenytoin
**Pramipexole** Mirapex^®^ [85]	Parkinson disease, restless legs syndrome	Nonergot dopamine agonist with specificity for the D_2_ subfamily dopamine receptor. Additionally, it binds to D_3_ and D_4_ receptors/Anti-parkinson agent, dopamine agonist	NA	>10%: orthostatic hypotension, drowsiness, extrapyramidal reaction, insomnia, dizziness, hallucination, headache, restless leg syndrome, abnormal dreams, nausea, constipation, dyskinesia, asthenia, accidental injury	Parkinson disease, restless legs syndrome
**Pregabalin** Lyrica^®^, Lyrica CR^®^ [86]	Fibromyalgia, neuropathic pain associated with diabetic peripheral neuropathy or spinal cord injury, partial-onset seizures, postherpetic neuralgia	modulates calcium influx at the nerve terminals, thereby inhibits excitatory neurotransmitter release, may also affect descending noradrenergic and serotonergic pain transmission pathways from the brainstem to the spinal cord/Anticonvulsant, GABA analog	NA	Peripheral edema, Dizziness, drowsiness, headache, fatigue, weight gain, xerostomia, visual field loss, blurred vision	Hypersensitivity (e.g., angioedema) to pregabalin or any component of the formulation
**Quetiapine** SEROquel^®^ [87]	Bipolar disorder, unipolar MDD schizophrenia	Antipsychotic activity through a combination of dopamine type 2 (D_2_) and serotonin type 2 (5-HT_2_) antagonism/Second-generation (atypical) antipsychotic	MDD or nonpsychotic depression as an adjunctive therapy. Oral: 50 mg/day on days 1 and 2; increase to 150 mg/day on day 3. Usual dosage range: 150 to 300 mg/day.	>10%: increased blood pressure, orthostatic hypotension, tachycardia, decreased HDL-C, increased serum cholesterol, increased serum TG, weight gain	Hypersensitivity to quetiapine or any component of the formulation
			Doses up to 600 mg/day may be needed in psychotic depression. Nonpsychotic depression, monotherapy. Oral: 20 mg once daily; may be gradually increased up to 300 mg/day.		
**Risperidone** Perseris^®^, RisperDAL^®^, RisperiDONE^®^ [88]	Bipolar disorder, schizophrenia, bipolar mania, irritability associated with autistic disorder	High 5-HT_2_ and dopamine-D_2_ receptor antagonist activity/Antimanic agent, second-generation (atypical) antipsychotic	MDD and TRD, as an adjunctive therapy. Oral: 0.25 to 0.5 mg/day; may increase dose in increments of 0.25 to 1 mg/day every 3 to 7 days up to 3 mg/day. Usual effective dose: 1 to 1.5 mg/day.	Activating/sedating effects, angioedema, dyslipidemia, extrapyramidal symptoms, hematologic abnormalities, hyperglycemia, weight gain, hyperprolactinemia, neuroleptic malignant syndrome, orthostatic hypotension, QT prolongation, sexual dysfunction, temperature dysregulation	Hypersensitivity to risperidone, paliperidone, or any component of the formulation
**Rosiglitazone** Avandia^®^ [89]	Type 2 diabetes mellitus	Improving target cell response to insulin, a potent and selective agonist for PPARγ/Antidiabetic agent, thiazolidinedione	NA	>10%: increased HDL-C, increased LDL-C, increased total serum cholesterol, weight gain	Hypersensitivity to rosiglitazone or any component of the formulation; NYHA class III/IV heart failure
**Scopolamine (hyoscine)** Transderm Scop^®^, Buscopan^®^ [90]	Prevention of nausea and vomiting associated with motion sickness, recovery from anesthesia, and surgery	Blocks the action of acetylcholine; increases cardiac output, dries secretions, antagonizes histamine and serotonin/anticholinergic agent	NA	>10%: drowsiness, dizziness, xerostomia	Hypersensitivity to scopolamine, other belladonna alkaloids, or any component of the formulation, narrow-angle glaucoma
**Statins, for example,****Lovastatin** Altoprev^®^, Mevacor^®^ [91]	Adjunctive therapy to diet to reduce elevated total cholesterol, LDL cholesterol, Apo B and TG and to increase HDL-C in patients with primary hypercholesterolemia	competitively blocking the active site of HMG-CoA reductase/Antilipemic agent, HMG-CoA reductase inhibitor	NA	>10%: Increased creatine phosphokinase	Hypersensitivity to lovastatin or any component of the formulation, active liver disease or unexplained persistent elevations of serum transaminases, concomitant use of potent CYP3A4 inhibitors
**Telmisartan** Micardis^®^ [92]	Cardiovascular risk reduction, hypertension	Nonpeptide AT1 angiotensin II receptor antagonist/Angiotensin II receptor blocker, antihypertensive	NA	1% to 10%: intermittent claudication, chest pain, hypertension, peripheral edema, dizziness, fatigue, headache, pain, dermal ulcer, diarrhea, abdominal pain, dyspepsia, nausea, UTI, back pain, myalgia, upper respiratory tract infection, sinusitis, cough, flu-like symptoms, pharyngitis	Known hypersensitivity (e.g., anaphylaxis, angioedema) to telmisartan or any component of the formulation, concurrent use of aliskiren in patients with diabetes
**Valproic acid** [93]	Bipolar disorder, focal (partial) onset and generalized onset seizures, migraine prophylaxis	Increased availability and enhance the action of GABA, blocks voltage-dependent Na channels/Anticonvulsant, antimanic agent, histone deacetylase inhibitor	NA	>10: headache, drowsiness, dizziness, insomnia, pain, nervousness, alopecia, nausea and vomiting, abdominal pain, diarrhea, dyspepsia, anorexia, thrombocytopenia, infections, tremor, weakness, diplopia, visual disturbance, flu-like symptoms, accidental injury	Hypersensitivity to valproic acid, divalproex, derivatives, or any component of the formulation, hepatic disease, urea cycle disorders, mitochondrial disorders caused by mutations in mitochondrial DNA
**Vorinostat** Zolinza^®^ [94,95]	Cutaneous T-cell lymphoma	Inhibits HDAC enzymes, HCAC1, HDAC2, HDAC3, and HDAC6, which catalyze acetyl group removal from protein lysine residues/Antineoplastic agent, histone deacetylase inhibitor	NA	>10%: peripheral edema, fatigue, chills, dizziness, headache, alopecia, pruritus, hyperglycemia, weight loss, dehydration, diarrhea, nausea, dysgeusia, anorexia, xerostomia, constipation, vomiting, decreased appetite, proteinuria, thrombocytopenia, anemia, muscle spasm, increased serum creatinine, cough, fever, upper respiratory tract infection	Severe hepatic impairment
**Zinc** Galzin^®^, Wilzin^®^ [96]	Wilson disease	Induces production of the copper-binding protein metallothionein in enterocytes/Chelating agent	NA	Gastric irritation increased serum amylase, increased serum lipase, increased serum ALP	Hypersensitivity to zinc acetate or any component of the formulation

ADHD: Attention-deficit/hyperactivity disorder; ALP: Alkaline phosphatase; ALT: Alanine aminotransferase; ANA: Antinuclear antibodies; BDNF: Brain-derived neurotrophic factor; COX-2: Cyclooxygenase-2; CYP3A4: Cytochrome P450 3A4; GABA: Gamma-aminobutyric acid; HDAC: Histone deacetylase; HDL-C: High-density lipoprotein-cholesterol; HMG-CoA: 3-Hydroxy-3-Methyl-Glutaryl-CoA; LDL-C: Low-density lipoprotein-Cholesterol; MAO: Monoamine oxidase; MDD: Major depressive disorder; NA: Not available; NE: Norepinephrine; NMDA: N-Methyl-D-aspartate; NSAIDs: Nonsteroidal anti-inflammatory drugs; NYHA: New York Heart Association; PPAR: Peroxisome proliferator-activated receptor; TG: Triglyceride; TNF-α: Tumor necrosis factor-alpha; TRD: Treatment-resistant depression; US-FDA: The United States food and drug administration; UTI: Urinary tract infection.

**Table 4:** Clinical trials which study the effects of repurposed medications on MDD since 2015.

Treatment (Tₓ) Phase/Year First, Posted Ref	Dosage (Duration of Therapy)	Subjects/F/M Condition Groups	Study Design	Results	Non-Serious AEs (Treatment-Related)	Serious AEs (Treatment-Related)
General anesthetics						
Ketamine NA/2016 [97]	Experimental: 1 mg/kg IV ketamine for the duration of their ECT index course over 2–3 weeks Active comparator: 1 mg/kg of IV methohexital for the duration of their ECT index course over 2–3 weeks	52/NA/NA MDD	R, PG, DB	NA	NA	NA
Ketamine Early phase I/2015 [98]	Experimental: ketamine + TAU Active comparator: midazolam + TAU	9/NA/NA MDD, BP1 disorder, BP2 disorder, BP depression, suicidal ideation	R, PG, DB	NA	NA	NA
Ketamine Phase I/2017 [99]	Experimental: 4 ketamine infusions at 0.05 mg/kg—once weekly Active comparator: 4 infusions at 0.045 mg/kg—once weekly	25/NA/NA A major depressive episode, unipolar depression, BP depression	R, PG, QB	NA	NA	NA
Ketamine Phase IV/2016 [100]	Placebo: saline 0.9%, IV administration of 0.2 mg/kg or 50 mg Medication: ketamine (1st phase) IV administration of 0.2 mg/kg or 50 mg Medication: ketamine (2nd phase) additional 4 sessions (twice a week, 2 weeks) of 0.5 mg/kg over 40 min	45/NA/NA MDD	R, PG, QB	NA	NA	NA
Ketamine Phase IV/2015 [101]	Medication: ketamine 0.5 mg/kg over 40 min IV Other: MRI technology will be used before and after ketamine for patients with depression	16/8/8 MDD, anxious depression, depression	SG, OL	NA	None	Not reported
Ketamine NA/2021 [102]	Received IV ketamine in 2014–15 and will be evaluated in 5 years	11/NA/NA/ MDD, medication abuse, medications, relapse	Retrospective	NA	NA	NA
Ketamine NA/2017 [103]	Experimental: ketamine and 16 CBT sessions over 14 weeks Active comparator: ketamine and psychoeducation all sessions over 14 weeks	28/NA/NA MDD	R, PG, SB	NA	NA	NA
Ketamine Phase II/2018 [104]	Experimental: different dosages and regimens for MIJ821 Active comparator: ketamine infusion 0.5 mg/kg weekly Placebo Comparator: placebo infusion	70/35/35 TRD	R, PG, DB	NA	None	Hyperacusis, photophobia, vision blurred, dry mouth, nausea, fatigue, feeling of relaxation, gait disturbance, increased systolic blood pressure, decreased platelet count, dizziness, dysgeusia, headache, paresthesia, sciatica, somnolence, anxiety, depersonalization/derealization disorder, disinhibition, irritability, alopecia, pruritus
Ketamine Phase II/2016 [105]	Experimental: NRX-101 oral capsule + ketamine IV infusion + saline solution IV infusion Active comparator: lurasidone oral capsule + ketamine IV infusion + saline solution IV infusion	22/16/6 BP depression, suicidal ideation, suicide attempts	R, SG, QB	NA	None	Angina pectoris, tinnitus, vision blurred, diarrhea, dry mouth, coordination abnormal, dizziness, dysmetropsia, hypoesthesia, sedation, restlessness, depression, suicidal ideation, acute kidney injury, dry skin, hypertension
Antimanic agents						
Lithium Phase III/2015 [106]	Experimental: 40 mg ITI-007 administered orally as capsules once daily for 6 weeks Experimental: 60 mg ITI-007 administered orally as capsules once daily for 6 weeks Placebo Comparator: placebo administered orally as visually-matched capsules once daily for 6 weeks	529/NA/NA BD	R, PG, QB	NA	NA	NA
Risperidone 2011 [68,107]	Groups: olanzapine users, quetiapine users, risperidone users, all other antipsychotic users	17743/9692/8051 SCH = 475 MDD = 798 BP disorder = 270 Generalized anxiety disorder = 17 Other mental health disorders = 637 Unknown indication = 15546 Quetiapine = 4658 Olanzapine = 5856 Risperidone = 7229	Observational, cohort, retrospective	Comparison between quetiapine and olanzapine: quetiapine is associated with lower extrapyramidal symptoms and diabetes mellitus Comparison between quetiapine and risperidone: quetiapine is associated with lower extrapyramidal symptoms, but higher failed suicide attempt rates	NA	NA
Atypical antipsychotics						
Aripiprazole Phase II/2016 [108]	Oral tablet (2,5,10,15,20, or 30 mg) with an IEM QD (8 weeks)	49/31/18 BP1 (n = 22) SCH (n = 15) MDD (n = 12) T_x_ = 49	SG, OL, multi-center	NA	Rash, erythema, pruritus skin irritation, upper respiratory tract infection, sinusitis, headache, syncope, meniscus injury, sunburn, peripheral swelling, pain in extremity	Not reported
Quetiapine Phase III/2016 [109]	Active comparator: levomilnacipran ER 20–120 mg/d starting at 20 mg/d on days 1–2, 40 mg/d on days 3–7 in week 1, then between 40–120 mg/d during weeks 2–8 Active comparator: 1uetiapine XR 50 mg/d on day 1–2, 150 mg/d on days 3–7 in week 1, then between 150–300 mg/d during weeks 1–8 along with their current antidepressant	60/NA/NA MDD	R, PG, TB	NA	NA	NA
Quetiapine Phase II/2017 [110]	Experimental: 1 capsule of 20 mg JNJ-42847922 and 1 capsule of placebo once daily for 14 days. Then, JNJ-42847922 dose can be increased to 40 mg (2 capsules) until day 167 Active comparator: 1 capsule of quetiapine XR 50 mg along with 1 capsule of placebo once daily for 2 days, followed by 1 capsule of 150 mg along with 1 capsule of placebo once daily from day 3–14. Then, the dose can be increased to 300 mg (2 capsules) until day 167.	107/NA/NA MDD	R, PG, DB	NA	NA	NA
	Patients should continue to take their baseline SSRI/SNRI.					
Brexpiprazole Phase III/2018 [35]	Experimental: brexpiprazole, 2–3 mg/day, once daily for 6 weeks, oral administration Placebo Comparator: placebo, 2–3 mg/day, once daily for 6 weeks, oral administration	65/NA/NA MDD	R, PG, TB	NA	NA	NA
Dopamine agonists						
Pramipexole Phase IV/2014 [47,111]	Started at 0.125 mg BD PO and increased by 0.25 mg/day every 3–4 days to a target range of 1.0–2.5 mg/day (6 weeks)	51/25/26 MDD (n = 26) Healthy (n = 25) Healthy control patients did not receive study medication and only have baseline measures	Non-randomized, PG, OL	Symptom’s improvement	Nausea, heartburn, vomiting, ↑appetite, ↓appetite, diarrhea, constipation, somnolence, restlessness, insomnia, forgetfulness, sleep attacks, compulsive behaviors, impaired concentration, headache, lightheadedness, dizziness, dry mouth, fatigue, ↓ libido, sexual dysfunction, skin problems, sweating, impaired coordination, tremor, blurry vision, bruising	Not reported
Armodafinil Phase III/2011 [50,112]	Tablet, PO, QD in the morning, started at 50 mg/day; the dosage was increased by 50 mg/day on days 2 and 4, up to 150 mg/day. (8 weeks)	399/241/158 depressive episode despite maintenance therapy for BP1 disorder P = 199 T_x_ = 200	R, PG, DB, PC, multi-center	↓Severity of depression, ↓depressive symptoms, Improved functioning	Headache, nausea	Anxiety, BP1 disorder, depression, insomnia, suicide attempt, cholecystitis chronic, social stay hospitalization
Armodafinil Phase III/2010 [51,113]	Tablet, PO, QD in the morning, started at 50 mg/kg and titrated up in the first week to 150 or 200 mg/kg. Treatment with 200 mg/kg dose was discontinued via a protocol amendment. (8 weeks)	492/273/219 major depressive episode despite BP1 disorder maintenance therapy P = 230 T_x_ (150 mg/kg) = 232 T_x_ (200 mg/kg) = 30	R, PG, DB, PC, multi-center	↓ Depressive symptoms	Nausea, diarrhea, dry mouth, toothache, dyspepsia, headache, dizziness, insomnia, anxiety, suicidal ideation, nasopharyngitis, cough	Mania, psychotic disorder, suicidal ideation, pulmonary embolism, abortion spontaneous, accidental overdose, non-cardiac chest pain, coronary artery disease
Armodafinil Phase III/2010 [49,114]	Tablet, PO, QD in the morning, started at 50 mg/kg and titrated up in the first week to 150 or 200 mg/kg. Treatment with 200 mg/kg dose was discontinued via a protocol amendment. (8 weeks)	433/288/145 major depressive episode while taking at least 4 weeks of conventional maintenance medication P = 199 T_x_ (150 mg/kg) =201 T_x_ (200 mg/kg) =33	R, PG, DB, PC, multi-center	Improvement in depressive symptoms	Diarrhea, nausea, headache, migraine, insomnia, feeling jittery	Psychotic disorder, suicidal ideation, depressive symptom, aggression, acute hepatic failure, hepatitis acute
Modafinil with D-cycloserine (DCS) Phase III/2015 [7]	250 mg DCS before two weekly sessions, 100 mg modafinil before two weekly sessions	36/20/14/2 (transgender) MDD P = 11 T_x_ (modafinil) = 12 T_x_ (DCS) = 13	R, PG, DB, PC	NA	↑Energy/concentration, fatigue/low motivation	Not reported
Supplementation						
Omega-3 PUFA Not applicable/2018 [115]	One capsule (EPA 300 mg and 200 mg DHA) QD were given antidepressant (citalopram, escitalopram, paroxetine 1 tablet at night time). (12 weeks)	70/NA/NA Taking antidepressants P = NA T_x_ = NA	R, PG, SB, PC	NA	NA	NA
n-3 Polyunsaturated fatty acid Not applicable/2017 [116]	2 g of EPA and 1 g of DHA (12 weeks)	60/NA/NA MDD and cardiovascular disease P = NA T_x_ = NA	R, PG, DB, PC	NA	NA	NA
Zinc Not applicable/2020 [46]	Oral 30 mg zinc sulfate QD with SSRIs (8 weeks)	100/NA/NA MDD P = 50 T_x_ = 50	R, PG, DB, PC	NA	NA	NA
NMDA receptor antagonist						
Dextromethorphan hydrobromide Phase I/2016 [38]	Two 75 mg capsules PO, separated by 4 h	4/NA/NA MDD P = NA T_x_ = NA	R, PG, DB, PC	NA	NA	NA
Esketamine Phase III/2017 [117,118]	84 mg intranasal, twice a week (on days 1,4,8,11,15,18,22 and 25) along with the standard of care antidepressant treatment initiated on day 1 (4 weeks)	225/139/86 MDD and have suicidal ideation with intent but without psychotic features P = 112 T_x_ = 113	R, PG, DB, PC, multi-center	↓ MADRS total score (improved), improvement in the severity of suicidality measured by CGI-SS-r	Dizziness, headache, somnolence, dysgeusia, hypoesthesia, sedation, dizziness postural, nausea, constipation, vomiting, dissociation, insomnia, anxiety, ↑ blood pressure, blurred vision, vertigo	Suicidal depression, depression, suicide attempt, diabetic ketoacidosis
Esketamine Phase III/2017 [119,120]	84 mg intranasal, twice a week (on days 1,4,8,11,15,18,22 and 25) along with the standard of care antidepressant treatment initiated on day 1 (4 weeks)	227/136/91 MDD and having suicidal ideation with intent, but without psychotic features P = 113 T_x_ = 114	R, PG, DB, PC, multi-center	↓MADRS total score (improved), ↓ in the severity of suicidality measured by CGI-SS-r	Dizziness, dysgeusia, somnolence, headache, paresthesia, sedation, hypoesthesia, dizziness postural, nausea, vomiting, paresthesia oral, dry mouth, constipation, hypoesthesia oral, dissociation, anxiety, euphoric mood, depersonalization/derealization disorder, insomnia, vision blurred, diplopia, nasal discomfort, oropharyngeal pain, throat irritation, vertigo, ↑ blood pressure, hyperhidrosis, feeling drunk	Suicide attempt, suicidal ideation, depersonalization/derealization disorder
Esketamine Phase II/2019 [121]	Four low, medium, or high doses (three different groups) on days 1,4,8 and 11 via dry powder inhaler (2 weeks)	88/NA/NA TRD in the course of MDD P = NA T_x_ = NA	R, PG, DB, PC, multi-center	NA	NA	NA
Esketamine Phase I/2016 [122,123]	Treatment A: intranasal placebo on day 1 and oral placebo on day 2 Treatment B: intranasal placebo on day 1 and oral alcohol on day 2 Treatment C: 84 mg of intranasal esketamine on day 1 and oral placebo on day 2 Participants will receive one of the ABC, BCA, CAB, CBA, ACB, or BAC treatments in part A and intranasal placebo on day 1 and 84 mg of intranasal esketamine on days 4,8,11,15,18,22 and 25 in part B	23/16/7 MDD P = 20 T_x_ = 23	Part A: R, cross-over assignment, SB, PC, active-controlled, double-dummy, 3-period Part B: OL, PC, fixed sequence, single period	Improvement in overall depression scores in MADRS	Dissociation, dizziness, dysgeusia, paresthesia, fatigue, paresthesia oral, nausea, headache, feeling abnormal, nasal discomfort, vision blurred, somnolence, euphoric mood, tinnitus, diplopia, ↑ blood pressure, feeling drunk, dysarthria, feeling of relaxation, illusion, hypoesthesia, altered time perception	Not reported
Combined medications						
AXS-05 Phase III/2019 [40,124]	Oral tablets of 45 mg dextromethorphan and 105 mg bupropion, BID (6 weeks)	327/215/112 Moderate or severe MDD P = 164 T_x_ = 163	R, PG, DB, PC, multi-center	↓ MADRS total score, improvement in daily functioning, improvement in quality of life	Dizziness, nausea, headache, diarrhea, somnolence, dry mouth	Not reported
AXS-05 Phase II/2018 [39,125]	45 mg dextromethorphan and 105 mg bupropion BID and Bupropion as AC (6 weeks)	80/51/29 Moderate or severe MDD AC = 37 T_x_ = 43	R, PG, DB, active-controlled, multi-center	↓ MADRS total score, improvement in core symptoms of depression	Nausea, dizziness, dry mouth, ↓ appetite, anxiety	Not reported
AXS-05 Phase III/2019 [41,126]	Oral tablets of 45 mg dextromethorphan and 105 mg bupropion, BID (12 months)	876/380/496 MDD including TRD T_x_ = 876	SG, OL, multi-center	↓ Depression symptoms, improvement in functioning	Dizziness, nausea, headache, dry mouth, ↓ appetite	Not reported
Anticholinergic agents						
Scopolamine Phase IV/2017 [45]	Experimental: scopolamine (0.3 mg/1 mL, IM) BID; escitalopram (10 mg/d PO) QD Experimental: scopolamine (0.3 mg/1 mL, IM) QD; placebo (1 mL saline, IM) QD; escitalopram (10 mg/d PO) QD Placebo comparator: placebo (1 mL saline, IM) BID; escitalopram (10 mg/d PO) QD	66/NA/NA MDD	R, PG, QB	NA	NA	NA
Scopolamine Phase IV/2017 [44]	Experimental: participants will receive active medications scopolamine and naltrexone Placebo comparator: participants will receive placebo medication	14/NA/NA Depression	R, PG, DB	NA	NA	NA
Mucolytic agents						
N-acetyl cysteine Early phase I/2014 [127]	Experimental: sertraline and N-acetyl cysteine for 7 weeks Experimental: citalopram and N-acetyl cysteine for 7 weeks Experimental: existing depression medication treatment and N-acetyl cysteine for 7 weeks	10/NA/NA MDD	Non-randomized, SG, OL	NA	NA	NA
Antibiotics						
Minocycline Early phase I/2015 [128]	Experimental: minocycline 50 mg/day on week 1, 50 mg/BID on week 2, and 100 mg/BID weeks 3–8. For tapering, the dose will be reduced to 50 mg BID for a week and then stopped Placebo comparator: the number and appearance of the pills would be identical to those in the minocycline arm	115/NA/NA MDD	R, SG, QB	NA	NA	NA
Minocycline Phase II/2015 [129]	Experimental: minocycline and standard antidepressant treatment Placebo comparator: placebo and standard antidepressant treatment	168/NA/NA MDD	R, PG, TB	NA	NA	NA
Cycloserine Not applicable/2017 [130,131]	Group A: placebo + placebo: placebo 8 mm pill, single dose Group B: fludrocortisone + placebo: fludrocortisone Astonin H 0, 1 gm, single-dose + placebo 8 mm pill, single dose Group C: D-cycloserine + placebo: cycloserine 250 mg capsule, single-dose + placebo 8 mm pill, single dose Group D: fludrocortisone + D-Cycloserine: fludrocortisone Astonin H 0, 1 gm, single-dose + cycloserine 250 mg capsule, single dose	232/182/50 MDD = 116 Healthy = 116 P = 58 Group B = 58 Group C = 58 Group D = 58	R, PG, DB, PC	↑ Cognitive empathy in the group with stimulated mineralocorticoid receptor, ↓ cognitive empathy only for positive emotions in MDD patients with NMDA-R stimulation,	NA	NA
D-cycloserine Phase IIb/III/2016 [42,105]	Experimental: NRX-101 (D-cycloserine + lurasidone) oral capsule with fixed-dose (administered to subjects who respond to an intravenous infusion of ketamine—0.5 mg/kg administered over 40 min; on Day 0)ketamine intravenous infusion (Randomized administration of Ketamine or Placebo in a 3 to 1 ratio), saline solution intravenous infusion (Randomized administration of Ketamine or Placebo in a 3 to 1 ratio) Active comparator: Lusaridone in the same dosage as lurasidone in NRX-101 ketamine intravenous infusion (Randomized administration of Ketamine or Placebo in a 3 to 1 ratio), saline solution intravenous infusion (Randomized administration of Ketamine or Placebo in a 3 to 1 ratio) (6 weeks)	22/16/6 BP depression, suicidal ideas, suicidal ideation, attempted suicide Ketamine Followed by NRX-101 = 12 Ketamine Followed by Lurasidone = 5 Saline Followed by NRX-101 = 4 Saline Followed by Lurasidone = 1	R, SA, QB, multicenter	NA	Angina pectoris, palpitation, tinnitus, diplopia, vision blurred, diarrhea, vomiting, dyspepsia, abdominal distention, dry mouth, nausea, gastroesophageal reflux disease, fatigue, vulvovaginal candidiasis, thermal burn, wound, ↑weight, muscle spasm, coordination abnormal, dizziness, dysmetropsia, headache, hypoesthesia, akathisia, hypersomnia, lethargy, somnolence, sedation, dissociation, restlessness, euphoric mood, anorgasmia, depressed mood, depression, suicidal ideation, acute kidney injury, ejaculation delayed, dry skin, rash, hypertension	Not reported
Antidiabetic agents						
Metformin Phase I and II/2019 [132]	Experimental: fluoxetine 20 mg capsule once daily for 12 weeks + metformin 1000 mg XR tablet once daily for 12 weeks Placebo comparator: fluoxetine 20 mg capsule once daily for 12 weeks + placebo tablet once daily for 12 weeks	80/NA/NA MDD	R, PG, DB	NA	NA	NA
Ganglionic blocking agents						
Mecamylamine (TC-5214) Phase III/2012 [132,133]	Experimental: SSRI/SNRI + TC-5214, 0.1 mg BID SSRI/SNRI + TC-5214, 1 mg BID SSRI/SNRI + TC-5214, 4 mg BID Placebo comparator: SSRI/SNRI + placebo BID (8 weeks)	696/498/198 MDD with inadequate response to no more than one antidepressant P = 174 0.1 mg = 174 1 mg = 174 4 mg = 174	R, PG, DB, PC, multi-center	No significant difference between the two groups	Headache, dizziness, somnolence, dysgeusia, constipation, dry mouth, nausea, abdominal pain upper, diarrhea, vomiting, insomnia, agitation, nightmare, nervousness, fatigue, hyperhidrosis, influenza, UTI, ↑ alanine aminotransferase, nasopharyngitis, orthostatic hypotension, hypertension, hypotension, vertigo, ↑ appetite	Suicide attempt, suicidal ideation, depression, pneumonia, UTI, toxicity to various agents, food poisoning
Mecamylamine (TC-5214) Phase III/2010 [37,134]	Experimental: SSRI/SNRI + oral tablet TC-5214, 1–4 mg BID Placebo comparator: SSRI/SNRI + oral tablet placebo BID (8 weeks)	319/200/119 MDD or depression with inadequate response to no more than one antidepressant P = 160 T_x_ = 159	R, PG, DB, PC, multi-center	No significant difference between the two groups	Constipation, diarrhea, nausea, dry mouth, vomiting, headache, dizziness, somnolence, dizziness postural, abnormal dreams, insomnia, anxiety, agitation, nightmare, upper respiratory tract infection, nasopharyngitis, fatigue, ↑ weight, orthostatic hypotension, hyperhidrosis, musculoskeletal stiffness	headache
Mecamylamine (TC-5214) Phase III/2010 [132,134]	Experimental: SSRI/SNRI + oral tablet TC-5214, 1–4 mg BID Placebo comparator: SSRI/SNRI + oral tablet placebo BID (8 weeks)	295/189/106 MDD or depression with inadequate response to no more than one antidepressant P = 148 T_x_ = 147	R, PG, DB, PC, multi-center	No significant difference between the two groups	Constipation, nausea, dry mouth, diarrhea, abdominal distention, abdominal pain upper, headache, dizziness, somnolence, dizziness postural, sedation, tremor, orthostatic hypotension, hypertension, nasopharyngitis, sinusitis, influenza, fatigue, asthenia, insomnia, anxiety, back pain, vertigo, hyperhidrosis, vision blurred, ↑ appetite, ↑ aspartate aminotransferase	Uterine cancer
Mecamylamine (TC-5214) Phase III/2010 [132,133]	Experimental: SSRI/SNRI + TC-5214, 0.5 mg BID SSRI/SNRI + TC-5214, 2 mg BID SSRI/SNRI + TC-5214, 4 mg BID Placebo comparator: SSRI/SNRI + placebo BID (8 weeks)	641/355/276 MDD or depression with inadequate response to no more than one antidepressant P = 161 0.5 mg = 160 2 mg = 160 4 mg = 160	R, PG, DB, PC, multi-center	No significant difference between the two groups	Blurred vision, constipation, nausea, dry mouth, diarrhea, abdominal distention, vomiting, dyspepsia, flatulence, fatigue, pyrexia, asthenia, pain, nasopharyngitis, bronchitis, upper respiratory tract infection, sinusitis, ↑ aspartate aminotransferase, ↑ appetite, back pain, muscle spasms, muscle tightness, myalgia, headache, dizziness, somnolence, dizziness postural, akathisia, insomnia, nightmare, agitation, abnormal dreams, pollakiuria, orthostatic hypotension, hypertension	Major depression, suicidal ideation, clavicle fracture, rib fracture, scapula fracture, obstructive uropathy, renal failure acute, upper respiratory tract infection, benign prostatic hyperplasia, pulmonary fibrosis, convulsion
Mecamylamine (TC-5214) Phase III/2010 [135,136]	Experimental: SSRI/SNRI + oral tablet TC-5214, 1–4 mg BID Placebo comparator: SSRI/SNRI + oral tablet placebo BID (52 weeks)	813/566/247 MDD or depression with inadequate response to no more than one antidepressant P = 203 T_x_ = 610	R, PG, DB, PC, multi-center	No significant difference between the two groups	Constipation, nausea, dry mouth, diarrhea, abdominal pain, vomiting, abdominal pain upper, flatulence, abdominal distension, fatigue, seasonal allergy, nasopharyngitis, bronchitis, upper respiratory tract infection, sinusitis, influenza, UTI, gastroenteritis, gastroenteritis viral, contusion, muscle strain, ↑ weight, ↑ blood pressure, ↑ appetite, arthralgia, back pain, muscle spasms, musculoskeletal pain, neck pain, pain in extremities, myalgia, headache, dizziness, somnolence, dizziness postural, sedation, memory impairment, migraine, paresthesia, tremor, insomnia, abnormal dreams, agitation, anxiety, bruxism, cough, nasal congestion, oropharyngeal pain, wheezing, hyperhidrosis, rash, hypertension, orthostatic hypotension	Bradycardia, abdominal hernia, diverticulum, hemorrhoids, small intestinal obstruction, cellulitis, oral infection, pneumonia, brain contusion, cerebral vertebral fracture, contusion, facial bones fracture, fibula fracture, tibia fracture, toxicity to various agents, musculoskeletal chest pain, abortion spontaneous, alcohol withdrawal syndrome, psychotic disorder, suicidal ideation, suicide attempt, ovarian torsion, hypertensive crisis

AC: Active comparator; ADT: antidepressant therapy; AEs: Adverse events; BD: bipolar depression; BID: twice a day; BP: Bipolar; CBT: Cognitive behavioral therapy; CGI-SS-r: Clinical Global Impression–Severity of Suicidality—revised; DB: Double blind; DHA: Docosahexaenoic acid; EPA: Eicosatetraenoic acid; F: Female; FA: factorial assignment; IEM: Ingestible event marker; IM: intramuscular; ITI-007:Lumateperone LE: Leukocyte esterase; M: Male; MADRS: Montgomery-Asberg depression rating scale; MDD: Major depressive disorder; NA: Not available; NMDA: N-methyl-D-aspartate receptor; OL: Open label; P: Placebo; PC: Placebo-controlled; PG: Parallel-group; PO: by mouth; PUFA: Polyunsaturated fatty acid; QD: once a day; QB: Quadruple blind R: Randomized; SA: Sequential Assignment; SB: single blind; SCH: Schizophrenia; SG: Single group; SNRI: serotonin norepinephrine reuptake inhibitor; SSRI: Selective serotonin reuptake inhibitor; TAU: Treatment as usual; TB: Triple blind; TRD: Treatment-resistant depression; Tx: Treatment; WBC: White blood cell; UTI: Urinary tract infection.

## Data Availability

Not applicable.
